# The retinal environment induces microglia-like properties in recruited myeloid cells

**DOI:** 10.1186/s12974-019-1546-9

**Published:** 2019-07-20

**Authors:** Scott W. McPherson, Neal D. Heuss, Ute Lehmann, Heidi Roehrich, Md. Abedin, Dale S. Gregerson

**Affiliations:** Department of Ophthalmology and Visual Neurosciences, University of Minnesota, 2001 6th Street SE, LRB Room 314, Minneapolis, MN 55455 USA

**Keywords:** Retina, Microglia, Myeloid cells, Injury response, Origin

## Abstract

**Background:**

Microglia are essential to the development of the CNS and its homeostasis. Our prior findings suggested a niche model to describe the behaviors of retinal microglia. Here, we ask whether new myeloid cells recruited to the retina are constrained to resemble endogenous microglia morphologically and functionally.

**Methods:**

Use of CD11c^DTR/GFP^ transgenic mouse allowed identification of two niches of retinal microglia distinguished by being GFP^lo^ or GFP^hi^. We also used transgenic mice in which CX3CR1^+^ cells expressed YFP and were depletable following tamoxifen-induced expression of diphtheria toxin subunit A. We employed several ablation and injury stimulation protocols to examine the origin and fate of myeloid cells repopulating the retina. Analysis of retinal myeloid cells was done by microscopy, flow cytometry, and qRT-PCR.

**Results:**

We found that the origin of new GFP^hi^ and GFP^lo^ myeloid cells in the retina of CD11c^DTR/GFP^ mice, whether recruited or local, depended on the ablation and stimulation protocols. Regardless of origin, new GFP^lo^ and GFP^hi^ retinal myeloid cells were CD45^med^CD11b^+^Ly6G^−^Ly6C^lo^Iba1^+^F4/80^+^, similar to endogenous microglia. Following tamoxifen-induced diphtheria toxin ablation, myeloid cell repopulation differed in the retina compared to the brain and optic nerve. Stimulation of replacement GFP^hi^ cells was substantially attenuated in repopulating retinas after tamoxifen-induced diphtheria toxin ablation compared to control or radiation-ablated mice. In radiation bone marrow chimeric mice, replacement GFP^hi^ myeloid cells from the circulation were slow to repopulate the retina unless stimulated by an optic nerve crush injury. However, once stimulated, recruited GFP^hi^ cells were found to concentrate on injured retinal ganglion cells and were morphologically similar to GFP^hi^ cells in non-ablated control CD11c^DTR/GFP^ mice.

**Conclusions:**

The results support the idea that GFP^hi^ cells in the CD11c^DTR/GFP^ mouse, whether recruited or from resident microglia, mark a unique niche of activated retinal myeloid cells. We conclude that the retinal environment has a potent influence on the function, morphology, and proliferative capacity of new myeloid cells regardless of their origin, compelling them to be equivalent to the endogenous microglia.

**Electronic supplementary material:**

The online version of this article (10.1186/s12974-019-1546-9) contains supplementary material, which is available to authorized users.

## Background

Innate immune cells mount an early response to stress, injury, and infection in central nervous system (CNS) tissue, including the retina [1], and are important contributors to CNS development and homeostasis [2–4]. A substantial literature attributes a wide range of innate immune functions in the CNS to microglia, the tissue-resident myeloid cells of the CNS [5–8]. Tissue-resident macrophages are found in many tissues, and their origins continue to be studied. In the case of microglia, the embryonic yolk sac was found to be their origin [9–12]. Langerhans cells appear to be derived in part from the fetal liver [11, 13]. Most other tissue macrophages originate in bone marrow [14]. Regardless of their origin, these macrophages were recruited to and live in tissue niches in which multiple factors, including chemokines, cytokines, and corresponding receptors, play an important role in maintaining their presence and regulating their activity [15]. The vascular endothelial cells that mediate passage of cells from the circulation into tissues use combinations of chemokines and chemokine receptors [16–18], especially CCL2/CCR2, to facilitate entry of innate immune cells into tissues including the kidney [19], lung [20], gut [21, 22], brain [23, 24], and retina [25]. A growing literature describes populations of self-renewing tissue resident macrophages, and efforts to understand their origins and distinct functions [26]. For tissue-resident macrophages, such as microglia, that originate from embryonic sources no longer present in adult animals, identifying how these cells are maintained and/or replaced over the lifetime of the host has been a significant challenge.

Our interest in antigen presenting cells of the retina that could contribute to T cell-mediated autoimmune retinitis led us to examine the retina for cells possessing the antigen-presenting capabilities of dendritic cells. Using conventional dendritic cell markers, others found evidence for substantial numbers of dendritic cells in the inflamed retina associated with experimental autoimmune uveoretinitis, a model for the retinal autoimmune disease [27]. In the normal retina, a small number of candidate dendritic cells was found by staining for Dec-205 [28] and 33D1 [29]. We found that CD45^+^ myeloid cells isolated from normal retina functioned poorly as antigen presenting cells and also inhibited the antigen presenting activity of splenocytes in cocultures, yielding T cells with signs of anergy [30]. This led our studies to the CD11c^DTR/GFP^ mice which permits tracking and depletion of a candidate antigen presenting cell population via green fluorescent protein (GFP) and diphtheria toxin receptor (DTR) [31]. Using these mice, we reported that quiescent murine retina contains a small, local population of CD45^med^CD11b^hi^Ly6G^lo^GFP^hi^ cells that resembled dendritic cells in their ability to upregulate expression of MHC class II [32], and to process and present cognate antigen to antigen-specific naive T cells [32, 33]. Depletion of retinal GFP^hi^ cells in CD11c^DTR/GFP^ mice by intraocular administration of diphtheria toxin eliminated the local antigen-specific T cell response in the retina [34]. The number of GFP^lo^ microglia (CD45^med^CD11b^+^Ly6G^lo^GFP^lo^ cells) remaining after diphtheria toxin ablation was unchanged and did not support an Ag-specific T cell response. The numbers of retinal GFP^hi^ cells expanded in response to different types of retinal injury and stress, and the cells migrated to specific sites of injury [32, 35, 36].

More recently we reported in two injury models using CD11c^DTR/GFP^ mice that the expanded retinal GFP^hi^ cells were derived from microglia; appearing in response to the stress of cone photoreceptor degeneration in CD11^DTR/GFP^ × RPE65^−/−^ double transgenic mice [35] and in response to optic nerve injuries [37]. Results from ablation, parabiosis, fate-mapping, and optic nerve transection experiments showed that the GFP^hi^ cells in the retina were not derived from the circulation, but were rather derived from resident retinal microglia and/or microglia recruited from the optic nerve. In contrast to retinal microglia, many of the microglia from the optic nerve were also GFP^+^ and Ki67^+^ [37]. These findings suggested that there were two niches of retinal innate immune cells; a relatively stable microglia niche and a specialized niche of microglia, as represented by the GFP^hi^ cells in CD11c^DTR/GFP^ mice, that can function as dendritic cells and whose numbers dynamically expand and contract in response to stimuli. However, in contrast, we have observed that following certain severe injury protocols, such as those involving radiation bone marrow chimeras, retinal microglia were substantially replaced by circulating bone marrow-derived cells [32]. Given the recent recognition of non-parenchymal CNS macrophages in meninges, perivascular spaces, and choroid plexus that are distinct from microglia [38], the contaminating presence of non-parenchymal myeloid cells may confuse the analysis of either the putative parenchymal microglia or the GFP^hi^ myeloid cells we have found in the retina, optic nerve, and brain of CD11c^DTR/GFP^ mice. Importantly, the murine retina can be isolated without contamination by meninges and choroid plexus, simplifying the analysis of parenchymal microglia.

In this study, we explore the basis for the diverse observations regarding the origins of replacement myeloid cells and their responses in CNS tissue. Given the highly specialized and regulated environment of the retina, we asked whether bona fide microglia and their monocytic replacements acted similarly within the environment of the retina. To do this, we used two distinctly different means to ablate retinal microglia and asked how the myeloid cells that repopulated retina responded to stress. Using CD11c^DTR/GFP^ mice in conjunction with other transgenic mice, we found that the retinal microenvironment exerted a potent influence on the morphology and function of GFP^hi^ and GFP^lo^ cells derived from either CNS microglia or adult bone marrow as they occupied the retinal myeloid cell niches. Replacement retinal myeloid cells from the circulation were largely GFP^lo^ until stimulated by a retinal injury which induced GFP expression and migration to the site of injury. Since the vast majority of studies of CNS myeloid cells have been done in the brain, some of our experiments were done in parallel in the brain and optic nerve and revealed significant differences.

## Methods

### Mice

All mice were obtained from Jackson Laboratories. CD11c^DTR/GFP^ mice (#004509, B6.FVB-Tg (Itgax-DTR/EGFP)57Lan/J) express a chimeric membrane protein comprised of GFP and the DTR under control of a transgenic CD11c promoter [31]. Depletion of GFP^hi^ cells was done by administration of exogenous diphtheria toxin. CX3CR1^YFP-creER^ mice (#021160, B6.129P2(Cg)-*Cx3cr1*^*tm2.1(cre/ERT2)Litt*^/WganJ) have constitutive CX3CR1-restricted expression of yellow fluorescent protein (YFP) and a fusion protein of *cre* recombinase and estrogen receptor (ER). When crossed with mice capable of expressing diphtheria toxin subunit A (DTA) after removal of the *floxed* transcriptional stop sequence (ROSA^DTA^ mice, #009669, B6.129P2-*Gt(ROSA)26Sor*^*tm1(DTA)Lky*^/J), CX3CR1-expressing myeloid cells, including microglia in brain [39] and retina, are depleted by administration of tamoxifen, which induces expression of DTA within CX3CR1^+^ cells. Crossing CX3CR1^YFP-creER^ROSA^DTA^ mice with CD11c^DTR/GFP^ mice allowed the subset of GFP^hi^ cells among the YFP^+^ cells to be tracked and/or depleted with diphtheria toxin. Wild-type (wt) B6J (CD45.2^+^) mice, B6 mice congenic for CD45 (CD45.1^+^, #002014, B6.SJL-*Ptprc*^*a*^ *Pepc*^*b*^/Boy6), and CD45.2^+^ mice expressing GFP under control of a β-actin promoter (ACTb^eGFP^ mice, #003291, C57BL/6-Tg(CAG-EGFP)1osb/J), were also used. All mice were *rd8* negative [40]. Mice aged to 2 years showed no evidence of retinal degeneration. Mice were housed under cyclic light in specific pathogen-free conditions and were euthanized by CO_2_ inhalation.

### Tamoxifen and diphtheria toxin administration

Tamoxifen (Sigma, #T5648) was dissolved in olive oil at 30 mg/mL and injected inter-peritoneal*.* Dose and timing of the injections are described in the experiments with the mice harvested at the indicated time after the last tamoxifen treatment. Depletion of retinal GFP^hi^ cells was done with 1 μL (5 ng) injections of diphtheria toxin into the anterior chamber of the eye as described [32] with timing, number of doses, and harvest time described in the experiments.

### Optic nerve crush

The optic nerve crush (ONC) injury was performed as described [32, 36, 41]. DSAEK forceps (Ambler Surgical, #2197E) provided a controlled injury to the optic nerve, consistently limiting the loss of retinal ganglion cells to approximately 50% [37].

### Immunostaining of retinal flat mounts

Retinal flat mounts were prepared, stained, and analyzed as described [32]. Primary antibodies included rat anti-CD11b, clone M1/70, BD Bioscience; rat anti-Ki67, clone SolA15, eBioscience; and anti-β3-tubulin, ThermoFisher. Secondary antibodies (Invitrogen) included Alexa Fluor 594 donkey anti-rat IgG; or biotinylated anti-rabbit IgG and Alexa Fluor 488/streptavidin; or biotinylated anti-rat IgG and Alexa Fluor 350/Streptavidin. Cell nuclei were stained with 4′, 6-diamidino-2-phenylindole (DAPI, Vector Laboratories). GFP and YFP were detected by their fluorescence. For cell quantification, 8 individual 0.19 mm^2^ fields (4 central, 4 peripheral) per retina were examined. The total number of cells through the entire retina within a field or contained within the field of the indicated retinal cell layer was counted. Results expressed as a mean number of cells per field or total cells per retina which was calculated based on a retinal volume of 2.7 mm^3^.

### Flow cytometry of CNS tissue

Mice were euthanized, perfused, and the retinas removed as described [32]. Optic nerve and brain tissue were also obtained if indicated. The tissues were dissociated using a solution of 0.5 μg/mL Liberase/Blendzyme3 (Roche) and 0.05% DNase in calcium, magnesium-free Dulbecco’s phosphate-buffered saline, stained with indicated antibodies, and analyzed by flow cytometry as described [32]. For analyses that included anti-CD115, the Liberase/Blendzyme3 was omitted from the dissociation step. An entire retina comprised a single sample, thus each sample represents the entire population of immune cells in one retina. For brain, one hemisphere of the brain without the cerebellum was digested and then the entirety of a small aliquot equivalent to the volume of one retina was analyzed by flow cytometry. The optic nerve contains a high number of CD11b^+^ cells. Therefore, we analyzed the entirety of the 5 mm piece between the posterior pole of the eye and the optic chiasm. This volume of the optic nerve is about 9% the volume of the retina. Cell numbers from optic nerve samples were then normalized to retina and brain so that all analysis of CNS cells numbers is based on an equivalent volume of tissue. All antibodies were obtained from BD Bioscience or eBioscience.

### Retinal RT-qPCR

Retinas were removed as described above and mRNA was directly isolated using a μMACS mRNA isolation kit (Miltenyi Bioscience). RT-qPCR reactions for the indicated genes were run with an iQ5 thermocycler (Biorad). Relative expression compared to the average of two housekeeping genes (β-actin and GAPDH) was calculated using the ΔΔCT method.

### Generation and analysis of radiation bone marrow chimeric mice

Donor bone marrow was flushed from donor tibias and femurs using calcium, magnesium-free Dulbecco’s phosphate-buffered saline. The bone marrow was passed through a 70-μm mesh filter, and the red blood cells were lysed by addition of 0.17 M NH_4_Cl (10 min at 37 °C). The bone marrow was then washed and resuspended to 5 × 10^7^ cells/mL in the above phosphate-buffered saline. Recipient mice were given 1 × 10^7^ bone marrow cells via inter-peritoneal injection. Prior to bone marrow transfer, recipient mice were irradiated as indicated with 12–18 Gy of total body irradiation (^137^Cs, 2 × 6–9 Gy, with a 3-h rest interval) unless specified otherwise. Where indicated, head or body shielding was done with 2.5 cm of lead. The extent of chimerism was assayed by flow cytometry analysis of the retina and peripheral blood for CD45.1 and CD45.2.

## Results

### Section 1: analysis of retinal myeloid cells following tamoxifen-induced DTA ablation of CX3CR1^+^ cells

#### GFP^hi^ microglia in the retina were parenchymal and perivascular

Our previous studies of retinal myeloid cells in CD11c^DTR/GFP^ mice revealed that they contained a small GFP^hi^ subpopulation of CX3CR1^+^CD45^med^CD11b^hi^Ly6G^−^Ly6C^lo^F4/80^+^Iba-1^+^ cells [32, 36]. To expand our analysis of their niche, origin, and function under other conditions, we crossed CD11c^DTR/GFP^ mice with CX3CR1^YFP-creER^ mice. In these double transgenic mice microglia of the CNS, including the retina, express YFP with a subpopulation of these cells in the retina also expressing GFP (Additional file 1: Figure S1A–C). In addition to their parenchymal niche, primarily in the inner and outer plexiform layers of the retina, some retinal GFP^hi^ cells were also observed to be perivascular in both naïve and day 7 post-ONC retina (Additional file 1: Figure S1D-F). Retinal perivascular macrophages have been described by others [42–45]. We found that they were often GFP^hi^ cells in the CD11c^DTR/GFP^ retina. The GFP^hi^ perivascular macrophages found after an ONC might be interpreted as showing their origin in the circulation, but our previous studies with parabiosis showed that recruitment into retina post-ONC was rare [37].

#### Contrasts in short-term repopulation of myeloid cells in CNS tissue after tamoxifen-induced depletion by DTA expression in CX3CR1^+^ cells

**Fig. 1:**
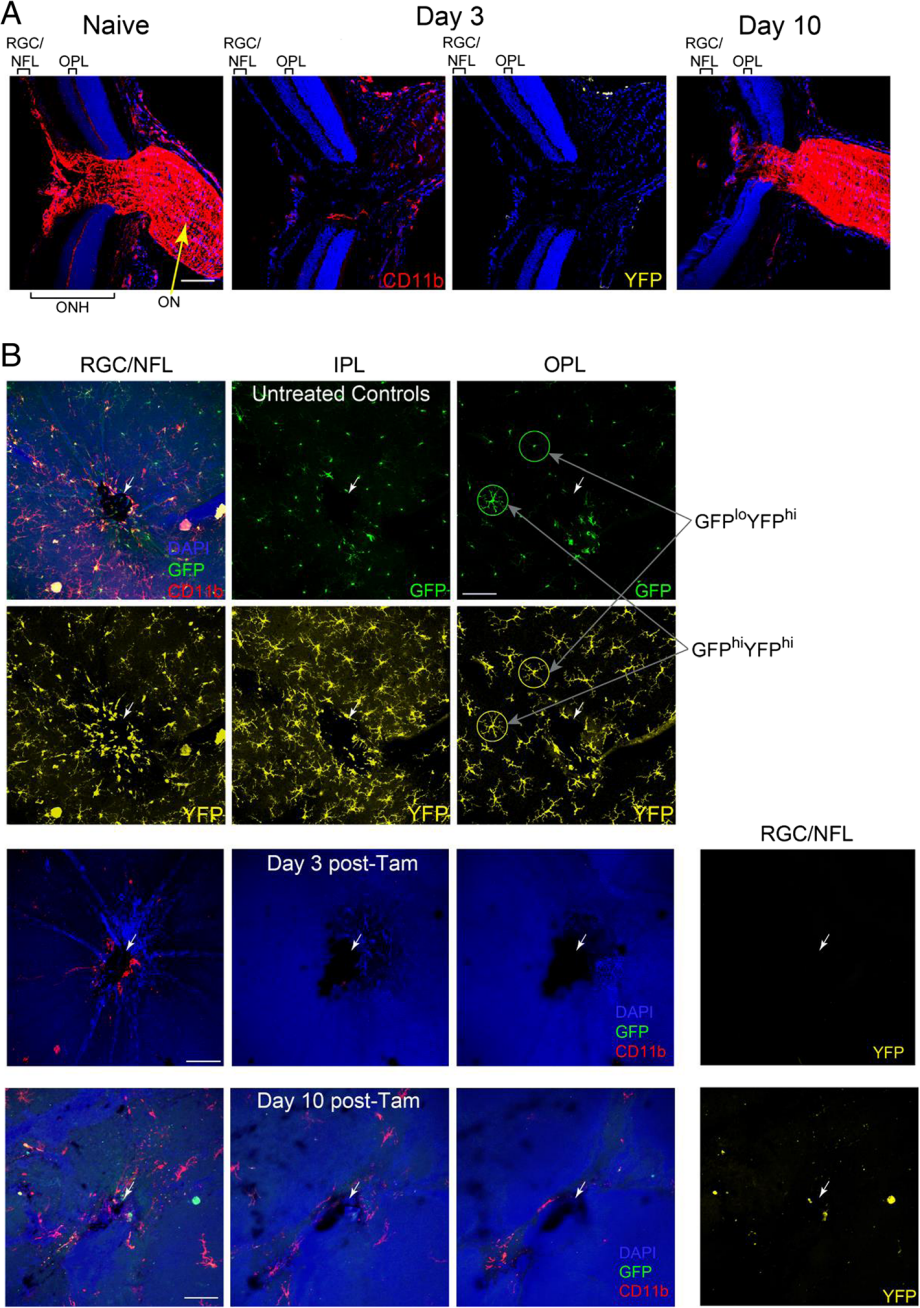
Repopulation of CD11b^+^YFP^+^ cells in ocular nervous tissue after tamoxifen-induced DTA ablation of CX3CR1^+^ cells. CD11c^DTR/GFP^:CX3CR1^YFP-creER^:ROSA^DTA^ mice were given 2.5 mg tamoxifen on three alternating days and analyzed at the indicated time after the last tamoxifen injection. **a** Sections through the posterior pole showing peripapillary retina, optic nerve head (OHN), and optic nerve (ON) of control mice (naïve, no tamoxifen) and mice at days 3 and 10 post-tamoxifen. **b** Flat mounts showing myeloid cells in the indicated layers of peripapillary retina from control mice and mice at days 3 and 10 post-tamoxifen. Layers include the retinal ganglion cells (RGC), the nerve fiber layer (NFL), inner plexiform layer (IPL), and outer plexiform layer (OPL). White arrows identify the optic nerve head. White scale bars are 100 μm. CD11b—red; GFP—green; YFP—yellow; DAPI—blue. Note that the somas of the YFP^hi^ cells are very bright and often bleed into the GFP channel. This is illustrated by the identification of two cells in the outer plexiform layer of panel B; one is GFP^hi^YFP^hi^ and the other is GFP^lo^YFP^hi^ (see also Fig. 3a). This is not an issue in flow cytometry which clearly distinguishes YFP and GFP

Others have recently shown that tissue-resident microglia in CNS and myeloid cells in some non-CNS tissues were derived and maintained independently of circulating monocytes and adult bone marrow progenitors [9, 46, 47]. We asked if those findings applied to the small population of GFP^hi^ cells in the retina. To ablate myeloid cells without radiation-induced damage, tamoxifen (to induce DTA expression) was administered to CD11c^DTR/GFP^:CX3CR1^YFP-creER^:ROSA^DTA^ mice to ablate CX3CR1^+^ cells (YFP^+^) cells. By 3 days of post-tamoxifen treatment, the optic nerve head and optic nerve were nearly devoid of YFP^+^ myeloid cells while a small number of CD11b^+^ cells remained (Fig. 1a). The retina was also well-ablated in all layers normally populated with YFP^+^ microglia (Fig. 1b). A few CD11b^+^ cells were found in around the optic nerve head in the retinal ganglion cell layer and nerve fiber layer at day 3 post-tamoxifen but they were not YFP^+^. By day 10 post-tamoxifen, the optic nerve was substantially repopulated (Fig. 1a) but the retina displayed limited recovery at days 3 and 10 (Fig. 1a, b). Recovery in the peripheral retina at day 10 was also minimal (not shown).

**Fig. 2:**
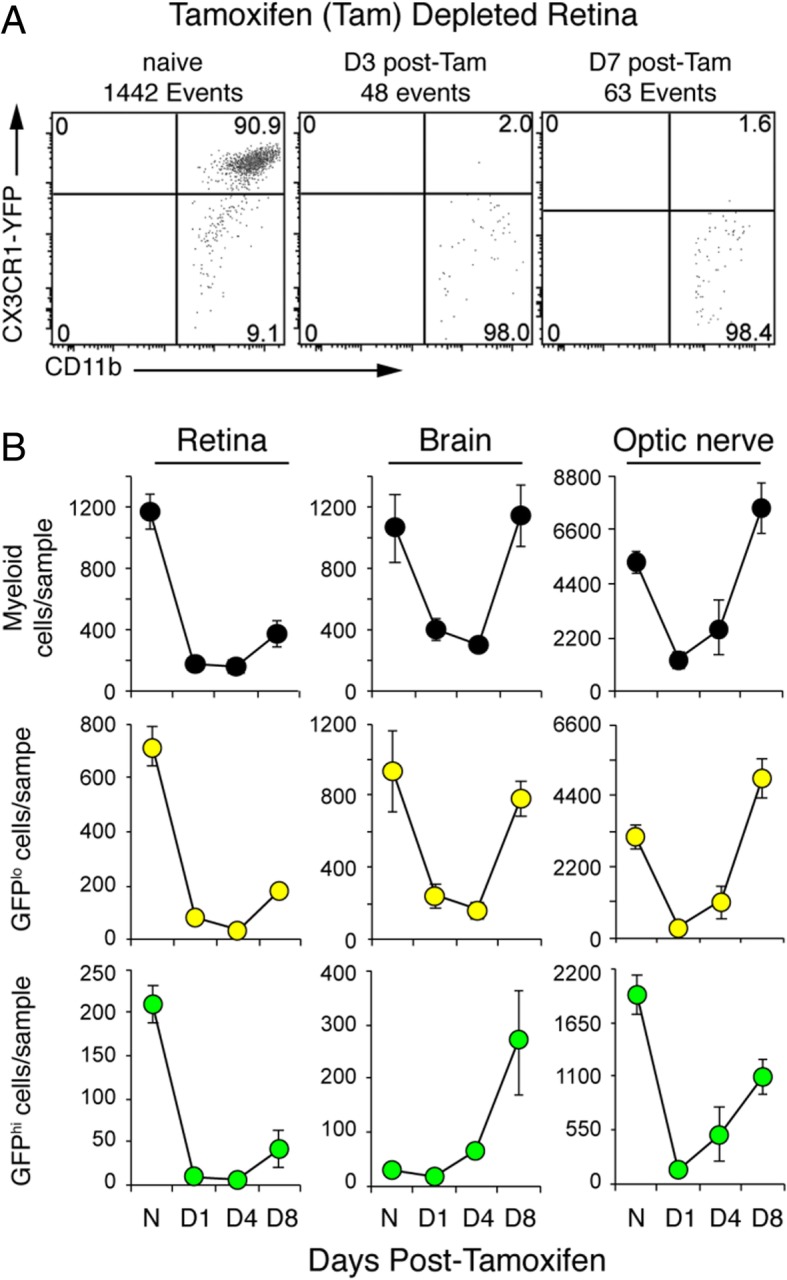
Repopulation of GFP^hi^ and GFP^lo^ microglia in CNS tissues of CD11c^DTR/GFP^:CX3CR1^YFP-creER^:ROSA^DTA^ mice after tamoxifen-induced DTA ablation. **a** Representative flow cytometry analysis of retinas confirming depletion of CX3CR1^+^ cells (2.5 mg tamoxifen on three alternating days) at 3 and 7 days after the last tamoxifen injection. Viable cells were gated on CD45^med^CD11b^+^Ly6G^−^ myeloid cells. **b** Number of myeloid cells counted by flow cytometry in retina, brain, and optic nerve from control mice (N, no tamoxifen) and tamoxifen-depleted mice at days 1, 4, and 8 post-tamoxifen. Cell number is based on equivalent tissue volumes and given as mean ± SD. Sample number at each time point ranged from 6 to 16 for retina, 3 to 9 for the brain, and 3 to 8 for optic nerve

Myeloid cell repopulation in the CD11c^DTR/GFP^:CX3CR1^YFP-creER^:ROSA^DTA^ tamoxifen-induced DTA-ablated retina, brain, and optic nerve was assessed by flow cytometry. Efficient ablation was confirmed at 3 days post-tamoxifen by the limited number of CD11b^+^CX3CR1-YFP^hi^ cells (Fig. 2a). Both GFP^lo^ and GFP^hi^ cells were rapidly and efficiently ablated, but striking differences in recovery of myeloid cells between the retina, brain, and optic nerve were found (Fig. 2b). The pace of recovery of myeloid cells in the brain after tamoxifen-induced DTA ablation was similar to findings in other studies [48–51]. Substantial differences were revealed by comparing GFP^lo^ myeloid cells versus GFP^hi^ myeloid cells in retina versus brain or optic nerve. The number of GFP^hi^ cells in normal brain was very low compared to the normal retina and optic nerve. In contrast to the retina, the brain was fully repopulated with myeloid cells by day 8 post-tamoxifen, and recovery included a supranormal number of GFP^hi^ cells. The number of GFP^lo^ myeloid cells per unit volume of the brain at day 8 post-tamoxifen was much higher than in the retina. The number of myeloid cells in the normal optic nerve is much higher per unit tissue volume than retina (Fig. 1a, naive and day 10 post-tamoxifen) or brain, with GFP^hi^ cells being especially abundant. However, following tamoxifen-induced DTA ablation, recovery in the optic nerve was more similar to the brain than retina in that it was rapid and substantially complete by day 8 post-tamoxifen. Overall, the recovery of both GFP^lo^ and GFP^hi^ myeloid cells in the retina lagged significantly compared to the brain and optic nerve.

#### Long-term repopulation of retina post-tamoxifen-induced DTA depletion was dominated by a transient increase in GFP^hi^ cells

**Fig. 3:**
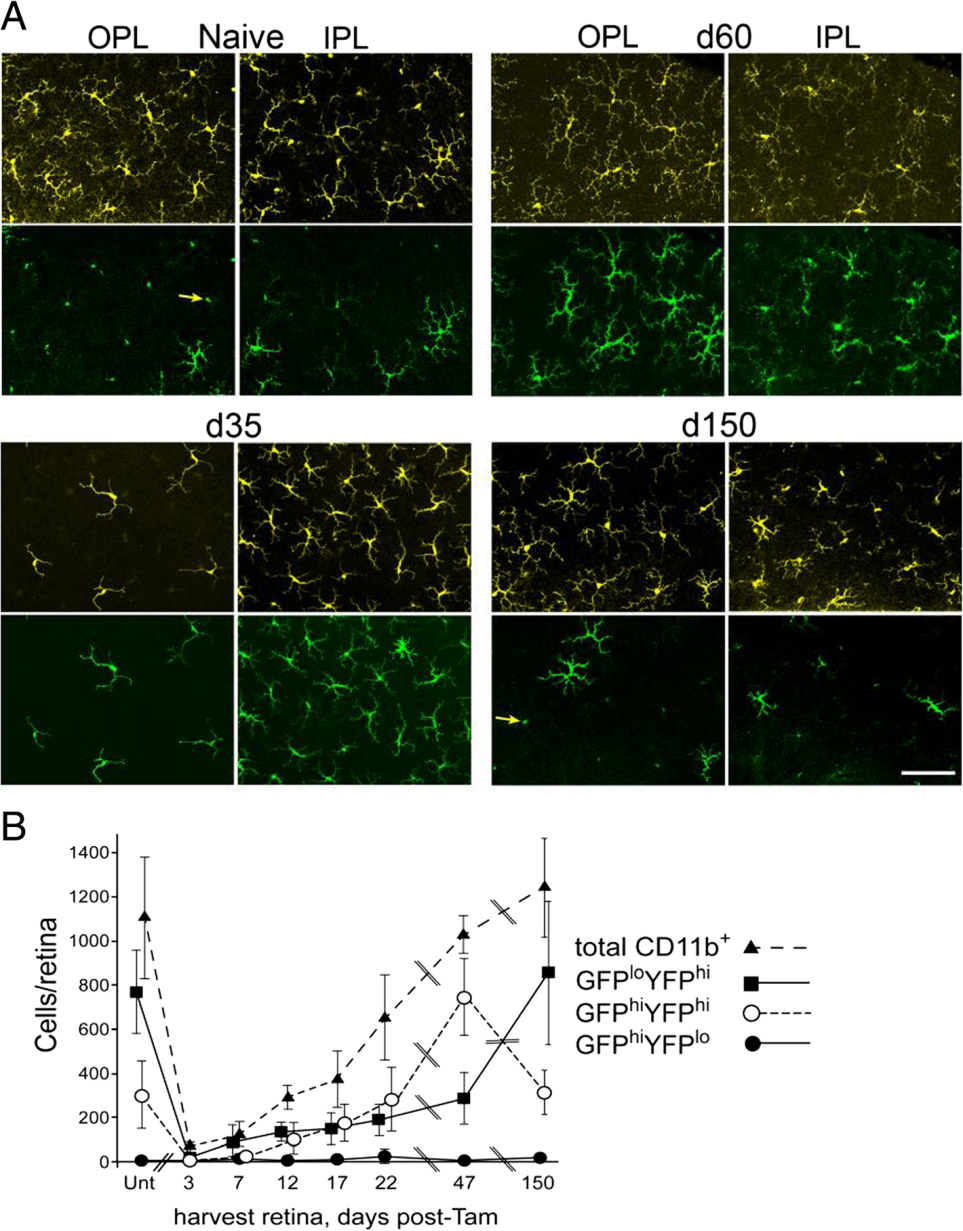
Repopulation of CD11c^DTR/GFP^:CX3CR1^YFP-creER^:ROSA^DTA^ retina after tamoxifen-induced DTA ablation of CX3CR1^+^ cells. Mice were given 3 mg tamoxifen on day − 2 and day 0 and harvested on the indicated days post-tamoxifen. **a** Microglia-like cells preferentially repopulate the inner plexiform layer (IPL) first and then the outer plexiform layer (OPL) after tamoxifen ablation. Yellow—YFP^hi^ cells; green—GFP^hi^ cells. Note again that cell bodies of YFP^hi^ cells are very bright, and often spill into the GFP channel, appearing as small green dots lacking dendrites in the GFP panels (examples marked by yellow arrows). Scale bar 100 μm. **b** Quantification by flow cytometry of the depletion and spontaneous repopulation of retinal CD11b^+^ (CD45^+^CD11b^+^Ly6G^−^) cells post-tamoxifen ablation. Cell number given as mean ± SD from control (UNT, no tamoxifen) mice or tamoxifen-treated mice. Four to eight retinas were harvested at each time point

While the short-term recovery of GFP^hi^ cells in the retina after tamoxifen-induced DTA ablation was reduced compared to the brain and optic nerve, we investigated the long-term recovery of retinal GFP^lo^ and GFP^hi^ cells. After tamoxifen-induced DTA ablation, repopulation of the retina by CD45^med^CD11b^hi^Ly6G^−^Ly6C^lo^ myeloid cells expressing GFP and/or YFP was followed for 150 days in CD11c^DTR/GFP^:CX3CR1^YFP-creER^:ROSA^DTA^ mice. Retinas were harvested at intervals for confocal microscopy and flow cytometry to assess the quantity and distribution of GFP^hi^ and YFP^hi^ cells in the retina during repopulation (Fig. 3). The inner plexiform was well repopulated with YFP^hi^ cells at 35 days post-tamoxifen (Fig. 3a) with the total number of CD11b^+^ cells in the entire retina differing little from naïve retinas at day 47 post-tamoxifen (Fig. 3b). In contrast to the normal retina, most of the YFP^hi^ cells were also GFP^hi^ at 35, 47, and 60 days post-tamoxifen (Fig. 3a, b). However, by 150 days post-tamoxifen the ratio of GFP^hi^ to YFP^hi^ cells had returned to levels similar to naïve retinas. The outer plexiform differed from the inner plexiform layer in that it was sparsely repopulated at 35 days and 60 days post-tamoxifen. As with the inner plexiform layer at these time points, most YFP^hi^ cells in the outer plexiform layer were also GFP^hi^. However, their morphology at days 35–60 post-tamoxifen lacked the extensive ramifications associated with microglia typical of the outer plexiform in a normal retina. By 150 days post-tamoxifen, myeloid cell morphology, total number, and ratio of GFP^hi^ to YFP^hi^ cells in the outer plexiform layer were similar to that of the naïve retina.

#### Tamoxifen-induced DTA-ablated retinas were refractory to injury stimulus

**Fig. 4:**
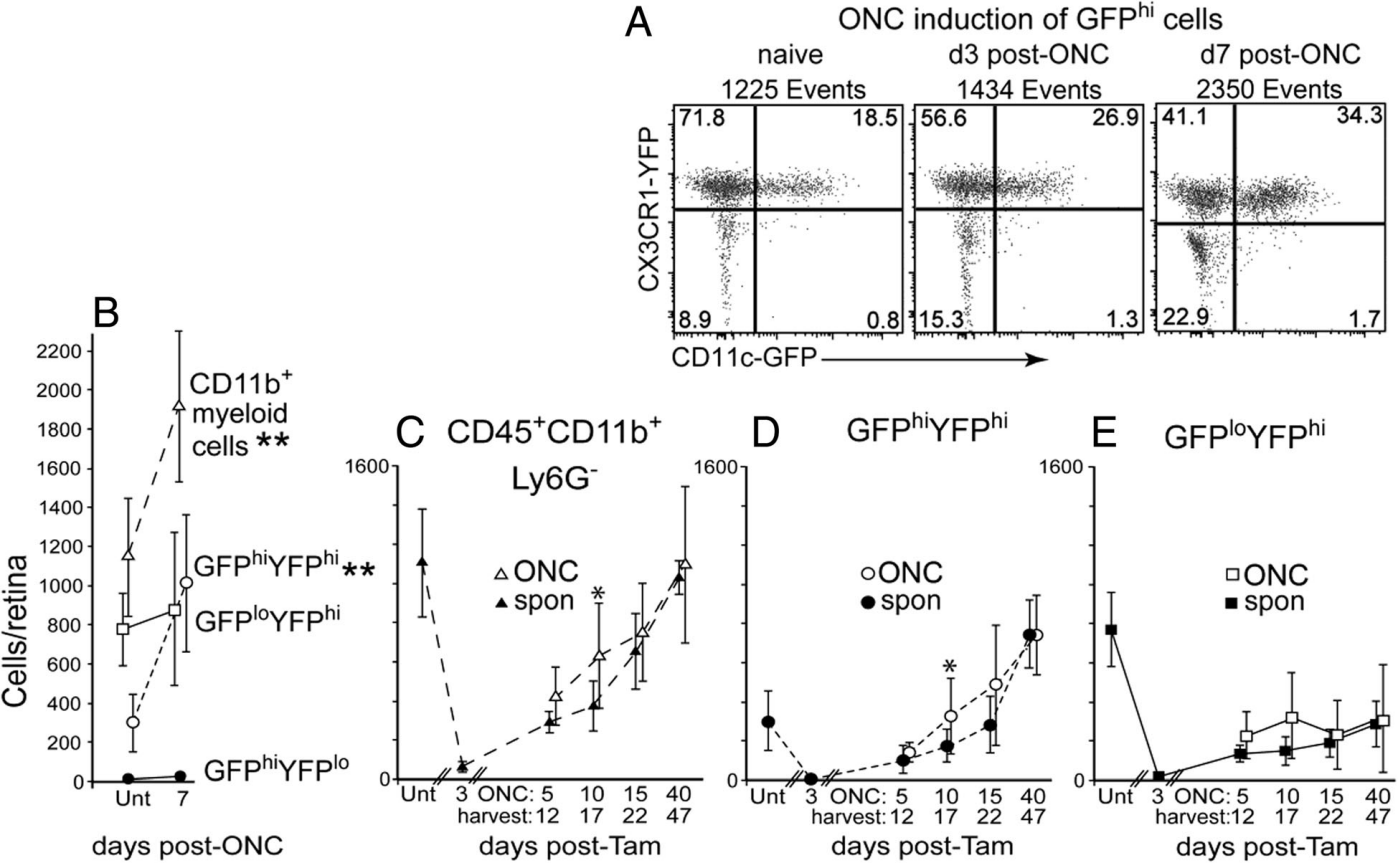
The injury response of the repopulating retina after tamoxifen-induced DTA depletion was diminished compared to controls. CD11c^DTR/GFP^:CX3CR1^YFP-creER^:ROSA^DTA^ mice were given tamoxifen (2.5 mg on three alternating days) or left untreated (Unt, no tamoxifen) and then given an ONC and harvested at the indicated day post-tamoxifen with retinas analyzed by flow cytometry. **a** Appearance of GFP^hi^ cells in substantial numbers at 7 days post-ONC in untreated (no tamoxifen) mice. **b** Quantification of the retinal myeloid cell response to an ONC in untreated mice. Open circles indicate GFP^hi^YFP^hi^ cells and open squares indicate GFP^lo^YFP^hi^ cells. **c–e** The injury response was strongly attenuated in tamoxifen-induced DTA-ablated retinas, even if performed after nominal repopulation. The analyzed cell populations include total CD45^+^CD11b^+^Ly6G^−^ cells (**c**), GFP^hi^YFP^hi^ cells (**d**), and GFP^lo^YFP^hi^ cells (**e**). Cell numbers are given as mean ± SD with 4–8 samples at each time point. Ipsi-ONC (tamoxifen plus ONC, open symbols) and spontaneously (spon) recovering retinas (tamoxifen only, closed symbols) indicated. *P < 0.05; **P < 0.01 for ONC versus non-ONC retinas

We previously showed that an ONC injury to retina of a CD11c^DTR/GFP^ mouse stimulated an increase in total retinal CD11b^+^ myeloid cells by 7–10 days that was largely due to the increase in GFP^hi^ cells. Hence, we asked if an ONC injury could enhance the speed and quantity of GFP^hi^ cell recovery in tamoxifen-induced DTA depleted CD11c^DTR/GFP^:CX3CR1^YFP-creER^:ROSA^DTA^ mice. Control experiments demonstrated that normal (no tamoxifen) CD11c^DTR/GFP^:CX3CR1^YFP-creER^:ROSA^DTA^ mice at 7 days post-ONC have an increased level of retinal CD11b^+^ cells that was primarily due to an increase in GFP^hi^ cells (Fig. 4a, b, open circles indicate GFP^hi^YFP^hi^ cells and open squares indicate GFP^lo^YFP^hi^ cells). Tamoxifen-induced DTA-ablated CD11c^DTR/GFP^:CX3CR1^YFP-creER^:ROSA^DTA^ mice were then given an ONC at either day 5, 10, 15, or 40 post-tamoxifen and their retinas analyzed 7 days post-ONC (Fig. 4c–e). Flow cytometric analysis comparing the total number of myeloid cells harvested from ONC (tamoxifen plus ONC) retinas versus non-ONC (tamoxifen only, spontaneous cell recovery) retinas showed a small, but detectable increase in the recovery of the total retinal CD11b^+^ population in ONC versus non-ONC retinas at day 17 (Fig. 4c), and for the GFP^hi^YFP^hi^ subpopulation of microglia at day 17 (Fig. 4d). Conversely, analysis of the GFP^lo^ microglia (GFP^lo^YFP^hi^) cell numbers showed no significant difference following an ONC from day 12 to day 47 (Fig. 4e). Although recovery from tamoxifen-induced DTA ablation based on the number of GFP^hi^ cells was complete, if not supranormal, at 40 days, an ONC at that time followed by analysis 7 days later gave no evidence of GFP^hi^ cell response to the ONC. Further, all of the ONC induced differences found in retinas post-tamoxifen were minimal in comparison to the ONC-induced responses generated in retinas without prior tamoxifen-induced DTA depletion (Fig. 4b vs c, d). These results are consistent with the repopulating GFP^hi^ subpopulation having less proliferative potential than the GFP^lo^ microglia.

#### CD115 and its ligands during recovery from tamoxifen-induced DTA depletion and injury

**Fig. 5:**
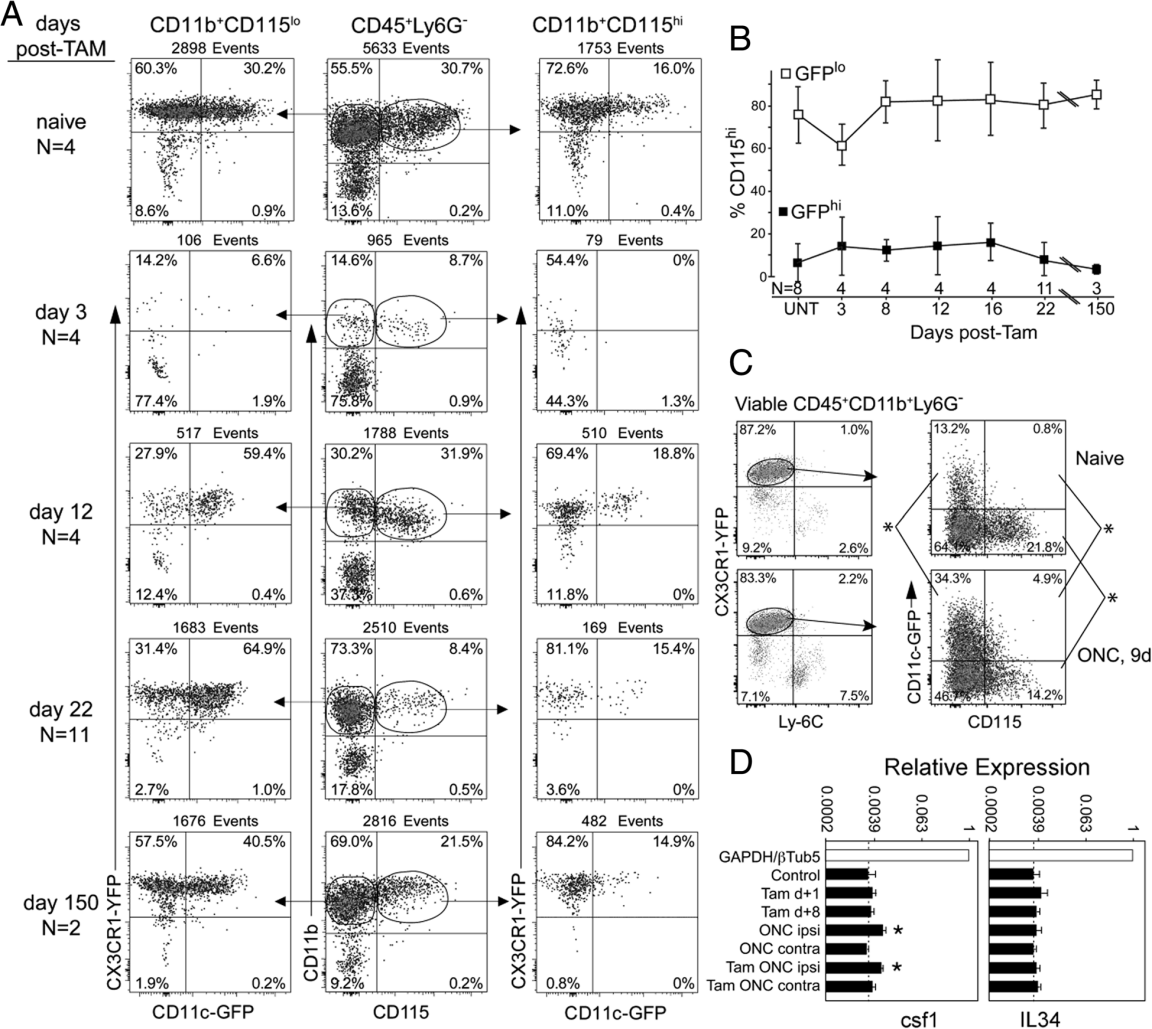
Properties of YFP^hi^ cells during recovery from tamoxifen-induced DTA depletion. CD11c^DTR/GFP^:CX3CR1^YFP-creER^:ROSA^DTA^ mice were given 2.5 mg tamoxifen on three alternating days and harvested at the indicated time after the last tamoxifen injection. **a** A substantial contribution to the repopulation of the depleted retina was made by GFP^hi^CD115^lo^ cells. Flow cytometry plots of the retinas were concatenated to assist visualization of the differences in CD115 expression on GFP^hi^ versus GFP^lo^ cells. **b** At all times, a greater proportion of the CD115^hi^ cells have the GFP^lo^ microglia phenotype (data shown as mean ± SD, *P* < 0.01 at each time point). **c** Following ONC, CD115 remained concentrated in the CD45^med^SSC^lo^GFP^lo^ population of myeloid cells. **d** RT-qPCR analysis for CFS1 and IL-34. mRNA was prepared from indicated retinas (tam = tamoxifen, ONC ipsi = injured retina, ONC contra = uninjured retina). The ONC was done 5 days post-tamoxifen with retinas harvested 10 days post-ONC. *P < 0.05 compared to all other conditions

Signaling via CD115 (CSF-1R) is required to develop and maintain CNS microglia based on observations that microglia numbers were reduced in the absence of its ligands, CSF-1, and IL-34 [52–57]. IL-34 is particularly required for maintenance of normal numbers of microglia [49, 57, 58]. Although GFP^hi^ microglia were prominent in the recovery from tamoxifen-induced DTA ablation, they were minimally responsive to the ONC injury. We have also observed that the numbers of GFP^hi^ cells in CD11c^DTR/GFP^ retina rapidly declined as the injured ganglion cells and axons were cleared post-ONC [36]. We asked if a reduced level of CD115 expression on GFP^hi^ microglia could be a unifying factor in these observations. In the naïve retina, and at all times post-tamoxifen, expression of CD115 was most closely associated with GFP^lo^ microglia rather than GFP^hi^ microglia (Fig. 5a). Most CD11b^+^CD115^hi^ cells were GFP^lo^ (Fig. 5a, right column) while there was always a greater number of CD11b^+^CD115^lo^ cells that were GFP^hi^ versus CD11b^+^CD115^hi^ cells (Fig. 5a, left column vs right column). Despite large changes in the frequency of GFP^hi^ cells during repopulation, the presence of GFP^hi^CD115^+^ cells was substantially lower than GFP^lo^CD115^+^ cells throughout repopulation (Fig. 5b). In the ONC injury model, CD115 expression was also associated with GFP^lo^ microglia (Fig. 5c). RT-qPCR of quiescent, ablated, and injured retina showed that expression of IL-34 was unchanged, while CSF-1 expression was modestly elevated post-ONC (Fig. 5d) regardless of ablation, consistent with the maintenance of a stable, CD115^+^ microglia niche. The results were consistent with the repopulating GFP^hi^ cells being unable to contribute to an ONC response despite their recovery to supranormal levels by 40 days. Reduced CD115 expression on GFP^hi^ microglia is also consistent with their transient presence in the retina following ONC injury compared to GFP^lo^ microglia and may characterize an important distinction between GFP^lo^ and GFP^hi^ microglia.

### Section 2: analysis of retinal myeloid cells in radiation bone marrow chimeric mice

#### Effects of radio-ablation on recruitment of retinal myeloid cells

**Fig. 6:**
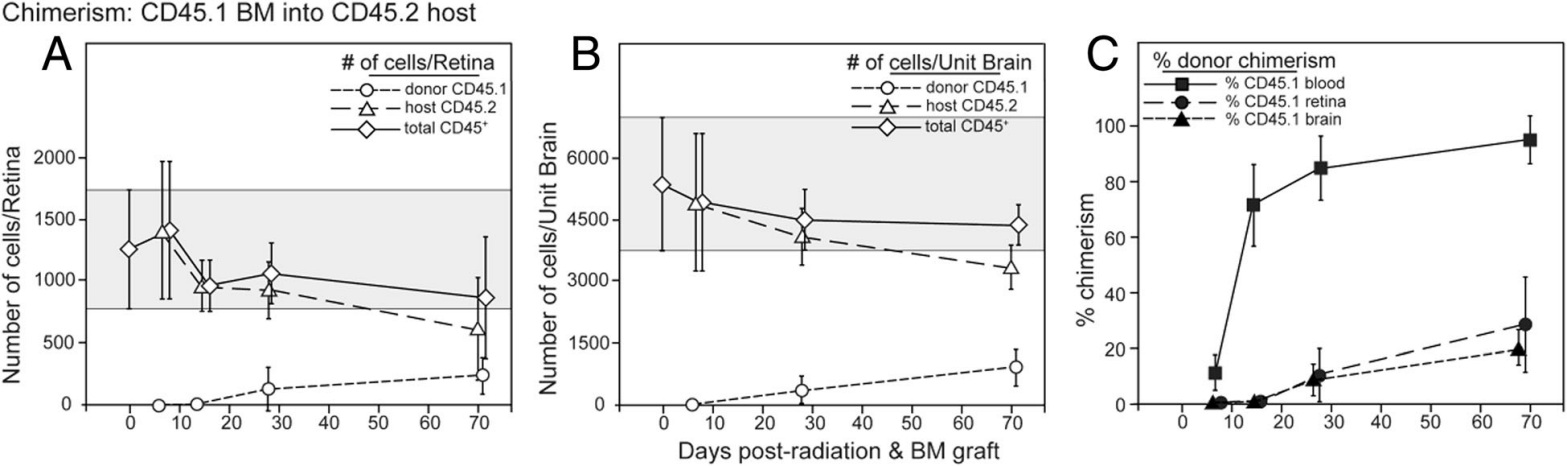
Recruitment of donor CD45^+^CD11b^+^ cells into the retina in radiation bone marrow chimeras. Chimeric mice received 2 × 6 Gy of radiation and were unshielded. **a** Flow cytometry analysis of chimerism in retinas of mice grafted with B6-CD45.1^+^ bone marrow into irradiated B6-CD45.2^+^ recipients assayed at the indicated time points. Overall number retinal CD45+ cells and the number of the host and donor cells is indicated. Cell numbers are given as mean ± SD. Gray shaded area indicates mean ± 1 SD of the total number of mononuclear cells in retina prior to radiation and bone marrow grafting. **b** Analysis of chimerism in the brain from the same mice as **a**. **c** Incorporation of donor bone marrow-derived cells expressed as percent donor chimerism. Cells were gated on CD45^med^CD11b^+^ cells

Recruitment of donor-derived cells into irradiated retinas in chimeric mice has been attributed to radiation damage with or without administration of a bolus of bone marrow progenitor cells [59–61]. Para-inflammation induced by whole-body gamma irradiation promoted CCL2-dependent chimerism in mice and replacement of retinal CD11b^+^ cells [60]. To assess the long-term effects of radiation plus bone marrow transfer on the repopulation or replacement of retinal microglia, we analyzed the chimerism of CD11b^+^ cells in the retina and brain-irradiated CD45.2 recipient mice grafted with CD45.1 bone marrow. Host CD45.2^+^CD11b^+^ cells in both retina and brain of the chimeric mice exhibited a similar, slow replacement by circulating, CD45.1^+^ donor bone marrow cells (Fig. 6a, b). The progression of retinal chimerism lagged well-behind chimerism in the blood (Fig. 6c), but the total count of host- and donor-derived cells in the retina and brain was relatively stable, consistent with the presence of a niche dependent on local production of CD115 ligands (IL-34 and CSF-1) [49, 62]. These results suggested that retinal microglia themselves could be progenitors and that they were nominally sensitive to radiation. The addition of head-shielding to the irradiation protocol substantially reduced the influx of donor bone marrow-derived cells, so that retinas harvested 70 days post-grafting contained only 4% donor cells compared to 28% donor cells in the CD45^med^CD11b^+^ population in unshielded mice (data not shown). This result is consistent with the hypothesis that the microglia niche in the head-shielded retinas remained occupied, reducing the opportunity for replacement by circulating myeloid or monocytic cells.

#### Retinal injury in chimeric mice led to rapid replacement of host microglia

**Fig. 7:**
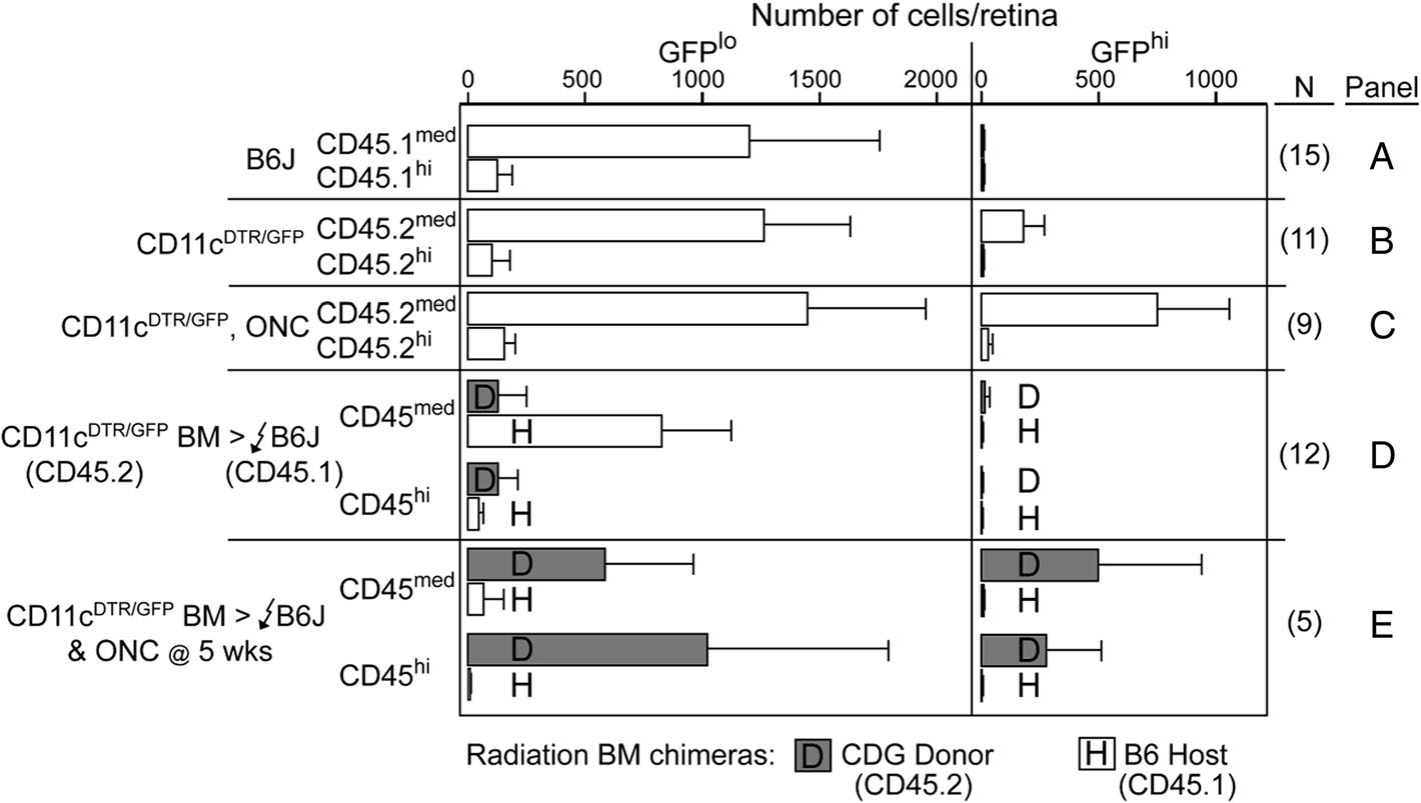
Irradiation of the retina compromises microglia survival leading to their replacement by donor bone marrow cells following ONC. **a**, **b** Analyses of retinal CD11b^+^CD45^+^GFP^lo^ and CD11b^+^CD45^+^GFP^hi^ cells in normal (non-chimeric, no ONC) B6-CD45.1 and CD45.2 CD11c^DTR/GFP^ mice. **c** ONC response in normal CD11c^DTR/GFP^ mice. **d**, **e** Analysis of CD45^+^ cells in the retinas of chimeric mice with or without ONC. Chimeric mice received 2 × 6 Gy of radiation and were unshielded. Retina was analyzed by flow cytometry for recipient (CD45.1) and donor (CD45.2) cells. ONC was done 5 weeks post-bone marrow transfer with retinas analyzed 7 days post-ONC. Cell numbers are given as mean ± SD with number of retinas in each group indicated

In a previous experiment, we established that retinal injury after tamoxifen-induced DTA ablation had little ability to enhance the replacement of retinal microglia (Fig. 4). Hence, we investigated whether the retinal injury would promote the replacement of retinal microglia following radiation-induced ablation and bone marrow transfer. Bone marrow from CD45.2 CD11c^DTR/GFP^ mice was grafted into irradiated B6-CD45.1-recipient mice. This allowed us to track the survival of recipient CD45.1 microglia, distinguish CD45.1 host retinal microglia from CD45.2 donor-derived myeloid cells, and distinguish recruited GFP^hi^ and GFP^lo^ cells. At 5 weeks post-bone marrow transfer, when retinal chimerism was still minimal, but blood chimerism was high (Fig. 6c), recipient mice were given an ONC. Retinas from injured and control groups were harvested 1 week later to assess recipient (CD45.1) and donor (CD45.2)-derived CD11b^+^ cells, expression levels of CD45, and GFP (CD45.2) expression (Fig. 7). After an ONC in non-chimeric CD11c^DTR/GFP^ controls, the responding cells were predominately CD45^med^GFP^hi^. Very few were CD45^hi^, which is consistent with a lack of recruitment of circulating cells (Fig. 7c). In chimeric mice that did not receive an ONC, host CD45.1^med^ cells were maintained at moderately reduced levels compared to non-chimeric control mice (Fig. 7d). However, these recipient mice contained only small numbers of donor-derived CD45.2^med^ and CD45.2^hi^ cells and very few were GFP^hi^ (Fig. 7d). In contrast, the retinas from chimeric mice receiving an ONC were predominantly repopulated by donor-derived CD45.2^med^ and CD45.2^hi^ cells, and many donor GFP^hi^ cells were found (Fig. 7e). The host microglia population (CD45.1^med^GFP^lo^ cells) was greatly reduced by day 7 post-ONC in the retinas of ONC-injured chimeric mice suggesting that the ONC is a sufficient stimulus for rapid turnover and recruitment of GFP^hi^ and GFP^lo^ myeloid cells into the irradiated retina (Fig. 7e). The percentage of donor-derived CD11b^+^CD45^+^ cells expressing GFP was much greater in chimeric mice receiving an ONC than in uninjured chimeric mice, indicative of CD45^+^ circulating precursors that entered the retina and upregulated their CD11c promoter/GFP reporter expression. The results show that bone marrow-derived cells repopulating a radiation-depleted retina were able to mount a cellular response to an ONC, unlike the cells that contributed to the repopulation of tamoxifen-induced DTA-ablated retina. Further, the recruited circulating myeloid cells upregulated GFP expression in response to the ONC injury.

#### Analysis of GFP^hi^ cells in radiation bone marrow chimeras and after diphtheria toxin ablation

**Fig. 8:**
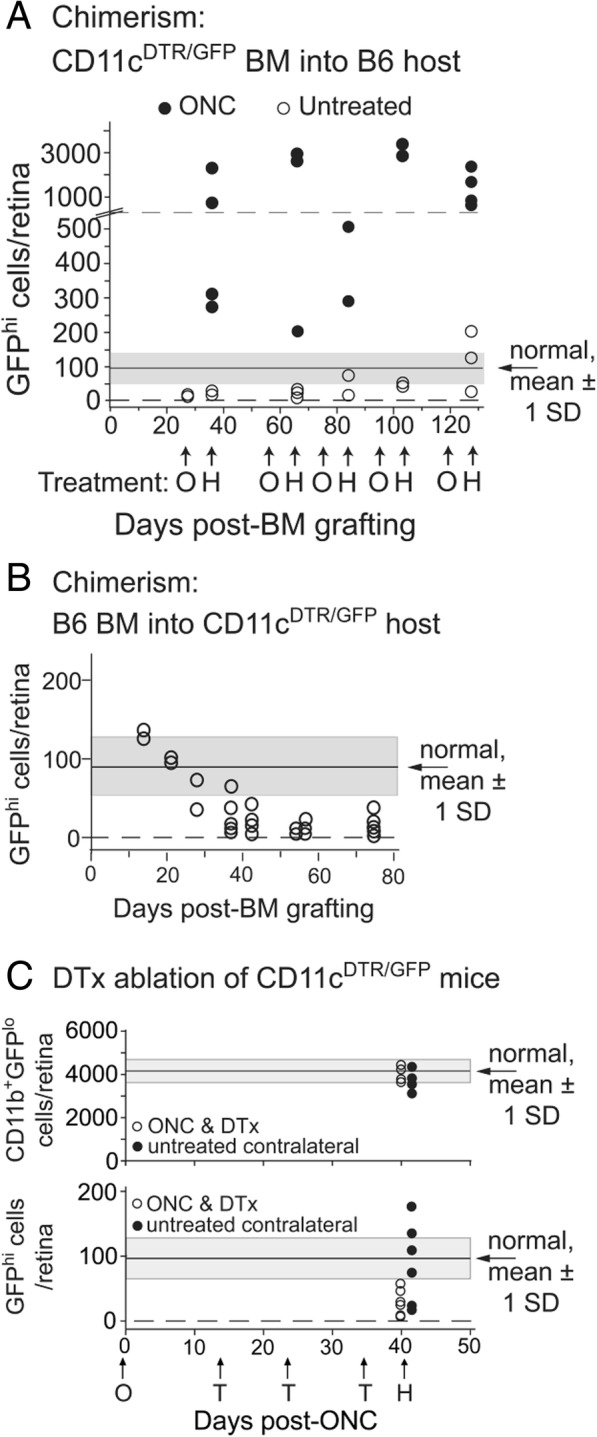
Analysis of GFP^hi^ cells in chimeric mice and after diphtheria toxin ablation. **a** Analysis of retinal GFP^hi^ cells in CD11c^DTR/GFP^ bone marrow (BM) into B6 recipients with or without ONC (O). Time of harvest (H) is indicated. **b** Survival of host retinal GFP^hi^ cells in CD11c^DTR/GFP^ recipients of B6 bone marrow. **c** Diphtheria toxin (DTx) treatment ablates only GFP^hi^ microglia. Mice were given a unilateral ONC and then that eye was treated with diphtheria toxin on the indicated days (T) then harvested. Contralateral eyes from the mice (no ONC, no diphtheria toxin) were harvested as controls. Chimeric mice received 2 × 6 Gy of radiation and were unshielded. Cell counts were obtained by staining retinal flat mounts for CD11b and then counting GFP^hi^ or GFP^lo^ cells. The average number of CD11b^+^GFP^hi^ or CD11b^+^GFP^lo^ cells (± 1 SD) in a normal CD11c^DTR/GFP^ mouse retina is indicated on the figures by the gray-shaded zone

The results above suggest that circulating cells (bone marrow-derived donor cells) are prominent in retinal myeloid (both GFP^hi^ and GFP^lo^ cells) repopulation following radiation ablation and ONC injury in CD11c^DTR/GFP^ mice. To further assay the ability to circulate precursors to contribute GFP^hi^ cells to the retina, we analyzed retinas of chimeric mice up to 128 days post-bone marrow transfer. CD11c^DTR/GFP^ bone marrow was transferred into irradiated B6 recipient mice. Groups of chimeric mice were given an ONC at 27, 57, 74, 94, or 118 days post-bone marrow transfer and their retinas analyzed 7 days later. Chimeric mice that did not receive an ONC were also analyzed on the indicated harvest date. In the absence of injury stimulus, circulating precursors from CD11c^DTR/GFP^ bone marrow slowly established a GFP^hi^ cell population in recipient B6 retinas that approached the level seen in normal CD11c^DTR/GFP^ retinas (Fig. 8a). However, at all time points, bone marrow-derived GFP^hi^ cells were found in substantial numbers in retinas of chimeric mice given an ONC. Reciprocal chimeras (B6 bone marrow into irradiated CD11c^DTR/GFP^ mice) exhibited a gradual loss of GFP^hi^ cells over time (Fig. 8b), suggesting replacement by donor cells incapable of expressing GFP. Together, these results show that radiation-induced ablation of retinal myeloid cells can induce a slow replacement by circulating cells. However, very much unlike replacement cells following tamoxifen-induced DTA ablation, a certain population of the myeloid cells (as represented by GFP^hi^) repopulating the retina after radiation are highly responsive to injury stimulation.

To confirm the unique nature of the retinal GFP^hi^ cells in CD11c^DTR/GFP^ mice, we compared the response of GFP^hi^ and GFP^lo^ microglia after an ONC to diphtheria toxin treatment. Mice were given a unilateral ONC followed by serial treatments of diphtheria into that eye. The treated retinas were harvested at the indicated times and compared to untreated retinas from the same mice (Fig. 8c). Analysis of retinal CD45^med^CD11b^+^ cells 45 days post-ONC showed that the number of GFP^lo^ cells was unaffected by either ONC or diphtheria toxin treatments. Conversely, there was a significant reduction in GFP^hi^ cells in response to diphtheria toxin even after an ONC. This data suggests, regardless of origin, GFP^hi^ cells represent a unique population of retinal myeloid cells.

#### Analysis of the injury response of donor-derived GFP^hi^ cells in chimeric mice and stratification of donor myeloid cells in retinal layers

**Fig. 9:**
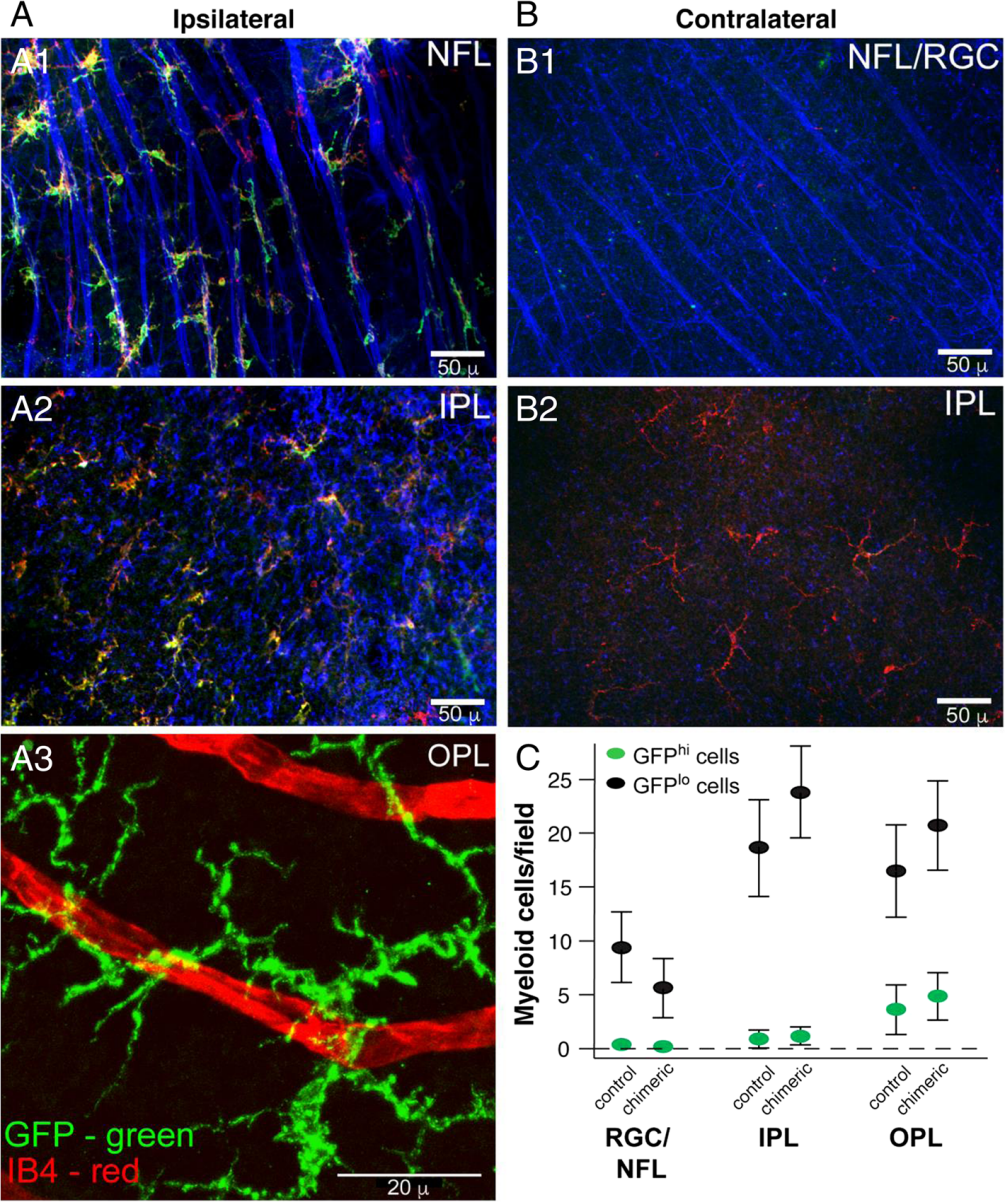
Injury response and long-term repopulation of donor-derived myeloid cells recruited into the retina mimics that of endogenous microglia. CD11c^DTR/GFP^ bone marrow was grafted into irradiated (2 × 6 Gy, unshielded) B6 mice. **a** Analysis of the ipsilateral (ONC injured) retina from a chimeric mouse given an ONC 6 weeks post-bone marrow transfer and analyzed 7 days post-ONC. **A1** Staining of retinal ganglion cells (RGC) and their axons in the nerve fiber layer (NFL) and underlying inner. **A2** and outer **A3** plexiform layers (IPL, OPL). All three layers analyzed were from the same microscopic field of the retina. **b** Same mouse as **a**. Analysis of the NFL/RGC and underlying IPL in the contralateral (uninjured) retina. For A1, A2, B1, and B2, green—GFP, blue—β3 tubulin, red—CD11b. For A3, green—GFP, red—isolectin B4 (IB4). **c** Analysis of myeloid cells in retinal layers in age-matched normal (control) CD11c^DTR/GFP^ mice and chimeric mice 180 days post-bone marrow transfer without ONC. Results are given as the mean number of cells per field ± SD, *n* = 4 (1 retina from four individual mice for each group), *P* > 0.05 control versus chimeric mice for each retinal cell layer

Previously, we documented the association of GFP^hi^ cells with the nerve fibers and soma of retinal ganglion cells post-ONC in non-chimeric CD11c^DTR/GFP^ mice, showing that approximately 90% of the microglia associated with the nerve fibers and soma were GFP^hi^ [37]. We then investigated whether this association would also occur in CD11c^DTR/GFP^ bone marrow into B6 chimeric mice. Analysis of ipsilateral retinal flat mounts from chimeric mice receiving a unilateral ONC at 6 weeks post-bone marrow transfer showed a close association between GFP^hi^ cells and nerve fibers (Fig. 9A1). Although most CD11b^+^ cells in the retinas of these chimeric mice are likely of donor origin (Fig. 7e), the fact that most (75.2% ± 13.4%, *N* = 6, analysis by cell counts on retinal flat-mounts) of the CD11b^+^ cells that aligned with nerve fibers were also GFP^hi^ confirmed their donor origin. Analysis of the underlying inner plexiform layer (Fig. 9A2, same field as Fig. 9A11) showed that GFP^hi^ cells were less prevalent among the CD11b^+^ cells which remained largely ramified despite their close proximity to the injured cells in the overlying retinal ganglion cell and nerve fiber layers. Donor-derived GFP^hi^ cells were also observed in the outer plexiform layer (Fig. 9A3). In contrast, the uninjured contralateral retinas contained very few donor GFP^hi^ cell in the nerve fiber layer (Fig. 9B1). The underlying inner plexiform layer contained donor CD11b^+^ cells but very few expressed GFP (Fig. 9B2). That contralateral retinas showed very little response beyond the basal repopulation is similar to the response observed in contralateral retinas from normal CD11c^DTR/GFP^ mice that received an ONC [32]. While migration of donor-derived myeloid cells in retinas of chimeric mice is slow in the absence of injury (Figs. 6 and 7), by 180 days post-bone marrow transfer the number and retinal location of donor myeloid cells were similar to the endogenous microglia (Fig. 9c). This migration, distribution, response, and morphology of GFP^lo^ and GFP^hi^ cells derived from donor myeloid cells in chimeric and chimeric/ONC injured mice resembled the microglia stratification and response seen in non-chimeric CD11c^DTR/GFP^ mice. These results suggest that the retinal environment strongly influenced the behavior of the recruited myeloid cells.

#### Donor-derived CD11b^+^ cells proliferated in retinas of radiation bone marrow chimeric mice

**Fig. 10:**
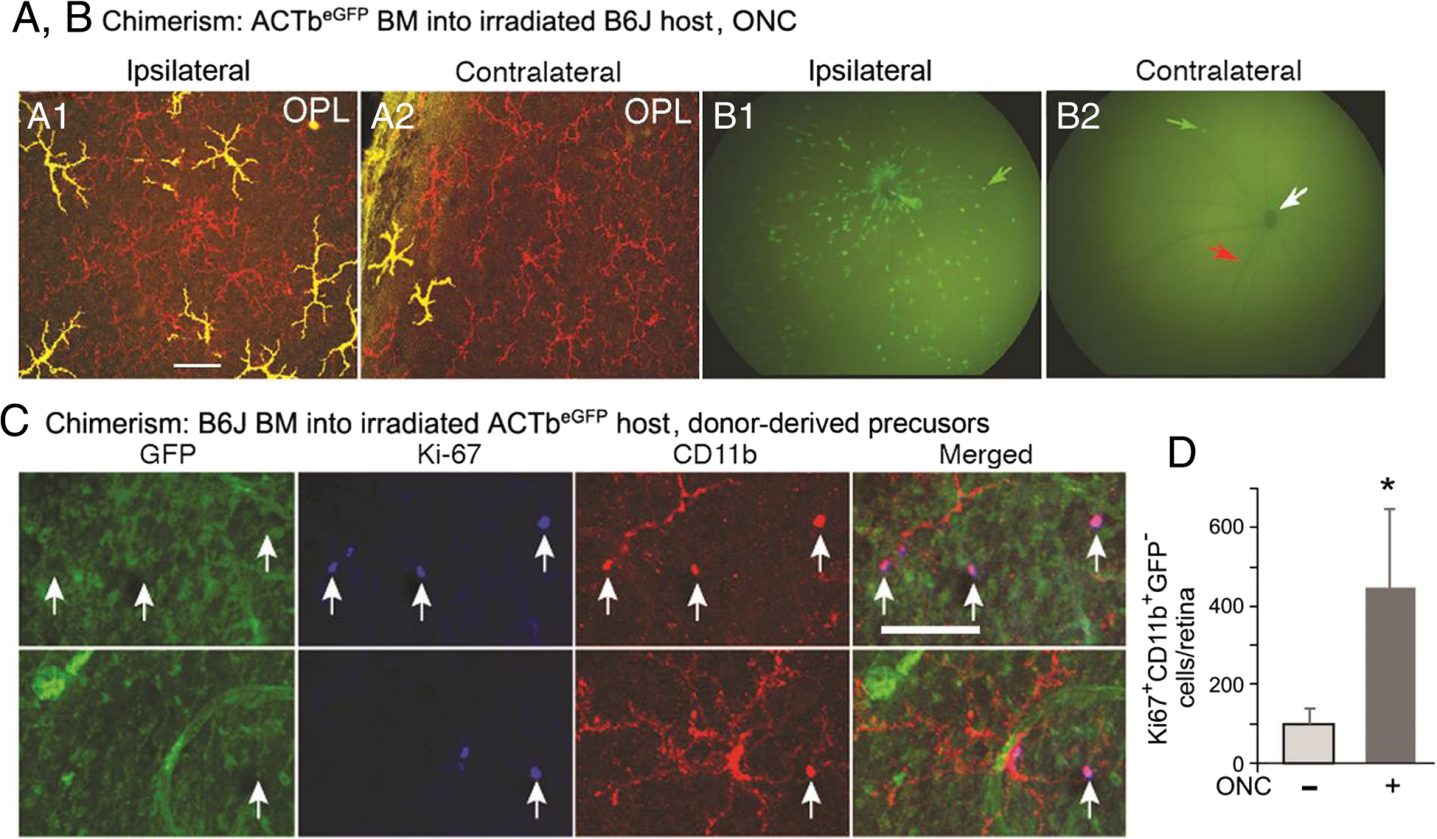
Myeloid cells recruited into the retina by an ONC injury in radiation-bone marrow chimeras acquired a microglia-like morphology and express the Ki67 cell proliferation marker. Chimeric mice (2 × 6 Gy, unshielded) were given a unilateral ONC 48 days post-bone marrow grafting and the retinas analyzed 7 days later. **a** ACTb^eGFP^ donor-derived GFP^+^ cells in B6 host retinas were visualized by fluorescence imaging of retinal flat mounts (**A1**) and by fluorescence retinal fundus imaging (**B1**) of a live mouse from ONC-injured retinas (ipsilateral). Analysis of the uninjured (contralateral) retinas (**A2**, **B2**) from the same mouse showed only rare donor-derived cells in the retina. Flat mounts were stained for CD11b (red) and analyzed for GFP (green). CD11b^+^GFP^+^ cells present as yellow. In the retinal fundus images, green arrow indicates a GFP^+^ cell, red arrow indicates a major blood vessel, and white arrow indicates the optic nerve head. **c** Retinal flat mounts of B6 bone marrow into ACTb^eGFP^ chimeric mice were analyzed for GFP (green), Ki67 (blue), and CD11b (red). Donor-derived proliferating mononuclear cells (GFP^−^Ki67^+^CD11b^+^ cells) were found in ipsilateral retinas 7 days post-ONC. **d** Counts of dividing Ki67^+^CD11^+^GFP^−^ cells in retinas with and without an ONC after bone marrow grafting. Cell number is given as mean ± SD, *n* = 6, *P < 0.01. Scale bars: (**a**) 50 μm; (**c**) 100 μm

Very few Ki67^+^ myeloid cells were found in retinas of radiation bone marrow chimeras made in B6 host mice given CD11c^DTR/GFP^ bone marrow prior to an ONC. Additional studies were done using ACTb^eGFP^ mice to detect the presence and proliferation of donor versus recipient ACTb^eGFP^ myeloid cells in the retina. Microscopy of retinal flat mounts from ACTb^eGFP^ bone marrow into B6 chimeric mice that had been given a unilateral ONC showed a number of ACTb^eGFP+^ cells in the retina (Fig. 10). The GFP^+^ cells were numerous in the ipsilateral (ONC injured) retina and exhibited a ramified, microglia-like morphology in the outer plexiform layer (Fig. 10A1). A very small number of GFP^+^ donor cells with a ramified morphology were found in the outer plexiform layer of the contralateral retina of the same mouse (Fig. 10A2). As evidenced by the yellow color of the cells in the fluorescence micrographs, all GFP^+^ cells were also CD11b^+^. Fluorescence photos of the retinal fundus showed the extensive infiltration of GFP^+^ cells in the ipsilateral retina post-ONC (Fig. 10B1), compared with the occasional GFP^+^ cell in the contralateral retina (Fig. 10B2). The presence of GFP^+^ cells in the retinas of these radiation bone marrow chimeras further confirmed the role of circulating precursors in the generation of retinal myeloid cells in response to injury in radiation bone marrow chimeric mice.

While the ACTb^eGFP+^ cells resembled microglia, their origin from donor bone marrow precludes their being bona fide microglia [9]. To confirm that proliferating cells in the chimeric retina were of donor origin, we analyzed reciprocal chimeric mice (B6 bone marrow into ACTb^eGFP^ recipients) for Ki67^+^ cells. Following an ONC, proliferating, donor-derived cells (GFP^−^Ki67^+^CD11b^+^) were readily found in recipient ACTb^eGFP^ retina, whereas dividing host cells (GFP^+^Ki67^+^CD11b^+^) were rare (Fig. 10c). Counts of GFP^−^Ki67^+^CD11b^+^ cells confirmed that ONC after bone marrow grafting significantly increases the rate of CD11b^+^ cell repopulation of irradiated recipient retinas via the proliferation of recruited myeloid cells within the retina (Fig. 10d).

#### Progenitor cells in the retina

Different paths to the repopulation of myeloid cells in retina versus optic nerve or brain were suggested by large differences in the rates of repopulation (Fig. 2). Others have shown a contribution of local progenitors to rapid repopulation of microglia following their depletion in the brain [49, 51, 63]. Evidence that local progenitor cells could contribute to retinal repopulation was sought by examining retinas by microscopy for progenitor cell markers (Additional file 2: Figure S2). The reliability of our staining technique was confirmed by detecting the presence of CD34^+^ and CD117^+^ cells in the bone marrow and fetal liver (Additional file 2: Figure S2D and F). CD34^+^ or CD117^+^ cells were not found in tamoxifen-induced DTA-ablated CD11c^DTR/GFP^:CX3CR1^YFP-creER^:ROSA^DTA^ mouse retinas at days 7 to 40 days post-tamoxifen. However, a small number of CD34^+^ cells were transiently found in the retinal ganglion cell layer of CD11c^DTR/GFP^ mice at days 4 to 10 post-ONC (Additional file 2: Figure S2A). GFP^hi^ cells were found in the vicinity of the CD34^+^ cells, with some cells expressing both CD34 and GFP (Additional file 2: Figure S2B and C). CD117^+^ cells were rare in quiescent retinas, but small groups were occasionally observed in the inner plexiform layer of the retina at day 4 post-ONC (Additional file 2: Figure S2E). However, analysis of quiescent and injured retinas failed to identify any cells that were double positive for any combination of CD34, CD117, and SCA-1, thus their identity as bona fide progenitors was uncertain.

## Discussion

The retinal microenvironment has a potent influence on the biology of its resident myeloid cells. New myeloid cells recruited to the retina whether from retinal progenitors, stores in adjacent CNS tissue, or from circulating monocytic cells, are constrained to substantially mimic the morphology and behavior of its resident, parenchymal myeloid cells, the microglia. Using the CD11c^DTR/GFP^ mouse which labels a subset of the microglia with GFP, the data in this study along with our previous reports has led us to propose that the retina maintains two niches of microglia. In the quiescent retina, the majority of microglia are CD45^med^CD11b^+^Ly6G^−^Ly6C^−^F4/80^+^Iba-1^+^ and do not express GFP (GFP^lo^). This niche of microglia is stable in number and carries out the functions associated with tissue macrophages that are necessary for the development and health of the retina. The second smaller niche of microglia is also CD45^med^CD11b^+^Ly6G^−^Ly6C^−^F4/80^+^Iba-1^+^ but is characterized by expression of GFP (GFP^hi^). This population of microglia dynamically expands and contracts in response to stimuli and can carry out the T cell activation functions of dendritic cells. It should be noted that all murine retinas have these two microglia niches but only with the CD11c^DTR/GFP^ mouse can they be distinguished. While there is controversy about the existence of GFP^hi^ cells in the retina of CD11c^DTR/GFP^ mice [64, 65], we find their presence to be obvious in the data we [32–37] and others [66, 67] have presented. Others have observed that myeloid cells recruited from the circulation often assume the appearance of microglia once inside retina [68, 69]. By exploring different strategies for repopulating the CD11c^DTR/GFP^ mouse retina, we found that recruited myeloid cells could also express GFP when activated by injury. While circulating myeloid cells recruited to the retina cannot be considered true or bona fide microglia, nonetheless, they look and act like microglia.

The retina has a number of unique properties that make it particularly well suited to study myeloid cell maintenance, replacement, and function in CNS tissue. First, the entire retina is easily removed, cleaned of adjacent tissue, and analyzed in its entirety as a unit. In this way, the total number (and changes to that number with treatment) of any given cell type in a retina can be analyzed directly. Second, the retina lacks meninges, and thus is a truer representation of neural parenchyma. While microglia occupy neural parenchyma of the brain, there is a substantial contamination of circulation-derived macrophages found in the perivascular/Virchow-Robbins space, subdural meninges, and choroid plexus [38]. Third, retina has a thin, flat structure that is amenable to full-thickness microscopy as flat mounts. Fourth, fluorescent markers can be visualized by non-invasive imaging through the pupil in vivo. Fifth, with a sufficient stimulus, the parenchyma can be repopulated with recruited myeloid cells able to take up long-term residence in the retina. We used strategies to ablate resident retinal myeloid cells and promote their replacement either from circulating myeloid cells or from CNS precursors and then compared the replacement myeloid cells to resident retinal microglia. While we observed differences in the dynamics and function of the replacement myeloid cells to resident microglia depending on the method of ablation and/or stimulation, we note that myeloid cells recruited from the circulation are very similar in function and phenotype to both the static and dynamic (as represented by GFP^lo^ and GFP^hi^ cells in the CD11c^DTR/GFP^ retina) resident microglia. We also note differences and similarities between the retina and other CNS tissue concerning myeloid cell repopulation after ablation.

One of the most striking differences between the retina and other CNS tissue was the kinetics of myeloid cell repopulation following tamoxifen-induced DTA ablation. In CD11c^DTR/GFP^:CX3CR1^YFP-creER^:ROSA^DTA^ mice, the short-term replacement of both GFP^lo^ and GFP^hi^ microglia in the retina significantly lagged compared to the brain and optic nerve. The rapid repopulation of brain microglia after ablation has been attributed in part to the activity of CD34^+^ and CD117^+^ progenitors [49]. However, we found minimal evidence for local progenitors in the retina. Even after injury we observed only a small, transient number of cells that were either CD34^+^, CD117^+^, or SCA-1^+^, and none of the individual cells stained for more than one marker, nor did any layer of the retina have cells positive for more than one marker. Thus, differences in putative progenitor cell numbers between brain and retina could account for the initially slow replacement in the retina. Another factor in CNS microglia repopulation could be the survival of residual microglia. A recent study proposed that after ablation, all microglia reappearing in the brain were derived from the few remaining survivors and not the circulation or de novo from resident progenitors [70]. Similar results have been reported in the retina [69], with the possibility that myeloid cells infiltrating from the adjacent ciliary body also contributing to retinal microglia repopulation [71]. While identifying sources of replacement microglia, these studies do not account for the different rates of microglia repopulation in various CNS tissues. If expansion of progenitors or residual microglia were replenishing the niches after ablation there should be evidence of cellular proliferation within the retina. However, using Ki67 as a marker for proliferation, the only instance in which we observe a significant number of retinal Ki67^+^ cells was with bone marrow transfer plus injury, and even then, the proliferating cells were of donor origin. We have also observed that an ONC can induce the appearance of numerous Ki67^+^ CD11b^+^ cells within optic nerve but few within the retina [37]. These results suggest that the retina differs from optic nerve and brain in that retina lacks the environment that supports myeloid cell proliferation in the absence of frank inflammation.

Repopulation of microglia in the retina and brain after tamoxifen-induced DTA ablation was characterized by a transient spike in GFP^hi^ cells. This was a rapid and relatively greater change in the brain (about 8-fold over background at day 8 post-tamoxifen) compared to the retina (about 3-fold over background at day 47 post-tamoxifen). While not accounting for the difference in timing in the GFP^hi^ cell response between retina and brain, we speculate that a lack of interplay between CD115 and its ligands CSF-1 and IL-34 contributes to its transient nature. CNS microglia numbers are reduced in the absence of CSF-1 and IL-34 [49, 52–58] suggesting the importance of these ligands in maintaining normal numbers of microglia. We observed that CD11b^+^GFP^hi^ cells had much lower levels of CD115 expression and that there was no change in either CSF-1 or IL-34 expression in tamoxifen-induced DTA depleted versus control retinas. Our results imply that CD115 and CSF-1/IL-34 levels were enough to replace and/or maintain GFP^lo^ microglia levels but could not support a sustained increase in GFP^hi^ microglia following tamoxifen-induced DTA ablation. The low levels of CD115 on GFP^hi^ cells would also account for their transient nature after ONC without microglia ablation despite a slight increase in retinal CSF-1 levels after injury.

Although concerns about the relevance and effects of irradiation have limited the use of radiation bone marrow chimerism strategies, we believe the approach has some merit. If microglia are self-renewing [46] or proliferating [72], or if there is a local microglia progenitor [49], they all may be radiosensitive, providing a strategy to induce their turnover and replacement by the grafted bone marrow. Repopulation of microglia in bone marrow chimeras differed from tamoxifen-induced DTA ablation in that brain and retina had a similar slow rate of replacement by donor myeloid cells. In tamoxifen-induced DTA-ablated brain and retina, the total number of CD11b^+^ cells declined sharply from normal levels before recovery whereas, in bone marrow chimeras, the total number of retinal CD11b^+^ cells remained close to normal with a gradual replacement of host microglia by donor myeloid cells. We found this to be the case for both retinal microglia niches as donor bone marrow cells slowly replaced both the resident GFP^lo^ and GFP^hi^ microglia. This is consistent with other reports that found the replacement of retinal resident microglia by donor-derived myeloid cells was an extended process in the absence of further manipulations [42].

The other major difference between tamoxifen-induced DTA and radiation-ablated retinal microglia was the ability of the replacement microglia, particularly the GFP^hi^ cells, to appear in response to injury. Attempts to stimulate recruitment or elevation of GFP^hi^ cell numbers by ONC were unsuccessful after tamoxifen-induced DTA ablation, even at 47 days post-tamoxifen when recovering retinas contained a supranormal level of GFP^hi^ cells and a subnormal level of GFP^lo^ microglia. In contrast, the outcome of injury was quite different if the ONC was performed in radiation bone marrow chimeric mice. Even when there was only a partial recovery of retinal GFP^hi^ cells after bone marrow transfer, an ONC simulated a significant response including a 90% reduction in recipient retinal microglia by 7 days post-ONC, and a large increase in donor-derived myeloid cells. A significant portion of the donor-derived cells, approximately 35%, were GFP^hi^. The overall magnitude of the ONC response was similar to that seen in a normal, non-chimeric CD11c^DTR/GFP^ retina, but was greater than 95% derived from circulating donor cells compared to less than 1% in normal mice. Further, approximately half of the retinal myeloid cells in chimeric mice after ONC were CD45^hi^, suggesting their recent influx into the retina. This vigorous, donor-derived GFP^hi^ cell response to ONC in chimeric mice could be observed to at least 128 days post-bone marrow transfer. We believe the ability to generate a GFP^hi^ cell response to ONC in radiation versus tamoxifen-induced DTA-ablated mice is 2-fold. First, in bone marrow chimeric mice, circulating cells recruited to the retina are CD115^hi^ and not expressing GFP, thus amenable to proliferation and/or expression of GFP upon injury stimulation. In contrast, after tamoxifen-induced DTA ablation, new retinal microglia are likely being recruited from the optic nerve and skewed compared to normal towards cells that are already CD115^lo^GFP^hi^ through at least 47 days post tamoxifen. Under these conditions, an ONC cannot enhance the rate of migration from the optic nerve.

These results are consistent with other reports demonstrating circumstances by which circulating monocytes [73], or other progenitor cells distinct from microglia could be recruited into the retina [68, 74, 75]. However, our results also suggest that regardless of the identity of the circulating precursor found to enter the retina in radiation bone marrow chimeric mice, their phenotype and function came to resemble the endogenous microglia. At 5 weeks, post-transfer retinal myeloid cells in B6 recipients given CD11c^DTR/GFP^ bone marrow were largely CD45^med^CD11b^+^Ly6G^−^Ly6C^−^F4/80^+^Iba-1^+^GFP^lo^ with a subpopulation being GFP^hi^. They were highly ramified and otherwise indistinguishable from microglia. Following ONC, there was a sharp increase in donor origin GFP^hi^ cells, and they formed a close association with the injured retinal ganglion cells and their axons similar to that seen in non-chimeric mice.

## Conclusions

Although the retina’s initial population of microglia is seeded from embryonic yolk sac progenitors through life even the quiescent retina may need to replace and replenish its endogenous microglia. This manuscript elucidates some of the basic mechanisms associated with retinal microglia renewal. Our work identifies two niches of retinal microglia that can be differentiated by GFP expression in transgenic mice expressing GFP from the CD11c promoter. Ablation of CNS microglia by tamoxifen-induced DTA expression induced a slower repopulation in the retina compared to other CNS tissue. The kinetics of this retinal repopulation could not be stimulated by injury and did not involve cells recruited from the circulation. In contrast, ablation by radiation-induced a retinal myeloid cell repopulation from circulating precursors which could be enhanced by injury. With either ablation, local progenitor cells were not an important factor in microglia repopulation. Our results suggest that replacement myeloid cells can come from multiple sources. However, regardless of the source, new myeloid cells recruited to the retina adapted the morphology and function of the endogenous retinal microglia in either niche. We conclude that the retina has a potent influence on the entry of mononuclear cells into the retina and on its myeloid cell occupants ensuring that essential, CNS-compatible functions are maintained.

## Additional files

Additional file 1: Figure S1 Confocal microscopy of GFP and YFP expression in retinas from CD11c and/or CX3CR1 promoters in transgenic mice. (A) GFP^hi^ cells present in the outer plexiform layer of naïve CD11c^DTR/GFP^ retina. (B) Detection of YFP^hi^ cells in the outer plexiform layer of naïve CX3CR1^YFP-creER^ retina. (C) Double transgenic CD11c^DTR/GFP^:CX3CR1^YFP-creER^ mice demonstrated YFP and GFP co-expression in some cells in the outer plexiform layer of the naïve retina. Note the cell in the lower right that is both YFP^+^ and GFP^+^. (D and E) Perivascular GFP^hi^ cells in naïve and day 7 post-ONC CD11c^DTR/GFP^ retinas. Green-GFP; red-isolectin B_4_. (F) GFP^hi^ cells were also found near small vessels in the vascular plexus in the inner plexiform layer. Green—GFP; red—CD11b; blue—isolectin B_4._ (DOCX 998 kb)

Additional file 2: Figure S2 Analysis of retinas for local progenitors following ONC. (A) Transient appearance of CD34+ cells in the retinas of non-ablated, non-chimeric CD11cDTR/GFP mice after ONC. CD34+ cell numbers (mean ± SD, *n* = 4) were determined by direct counts using fluorescence microscopy. (B, C) Immunofluorescence staining showing CD34+ cells in the ganglion cell layer of the retina at 4 days post-ONC. (D) Analysis of bone marrow cells for CD34 expression. (E) Analysis of a non-chimeric B6 mouse 4 days post-ONC revealed the occasional group of CD117+ cells in the inner plexiform layer. (F) Analysis of fetal liver cells for CD117 expression. (DOCX 1308 kb)

## Data Availability

Data and materials are available on request. Contact the corresponding author.

## Abbreviations

CNS: Central nervous system
DTA: Diphtheria toxin A subunit
DTR: Diphtheria toxin receptor
GFP: Green fluorescent protein
ONC: Optic nerve crush
YFP: Yellow fluorescent protein
