# Supplementary material for: The retinal environment induces microglia-like properties in recruited myeloid cells
Source: J Neuroinflammation. 2019 Jul 20;16:151. doi: 10.1186/s12974-019-1546-9 (PMC6642741; doi:10.1186/s12974-019-1546-9)
Supplement: Supplementary file 1 — Figure S1 Confocal microscopy of GFP and YFP expression in retinas from CD11c and/or CX3CR1 promoters in transgenic mice. (A) GFPhi cells present in the outer plexiform layer of naïve CD11cDTR/GFP retina. (B) Detection of YFPhi cells in the outer plexiform layer of naïve CX3CR1YFP-creER retina. (C) Double transgenic CD11cDTR/GFP:CX3CR1YFP-creER mice demonstrated YFP and GFP co-expression in some cells in the outer plexiform layer of the naïve retina. Note the cell in the lower right that is both YFP+ and GFP+. (D and E) Perivascular GFPhi cells in naïve and day 7 post-ONC CD11cDTR/GFP retinas. Green-GFP; red-isolectin B4. (F) GFPhi cells were also found near small vessels in the vascular plexus in the inner plexiform layer. Green—GFP; red—CD11b; blue—isolectin B4. (DOCX 998 kb) [file 12974_2019_1546_MOESM1_ESM.docx]

 **Additional File 1, Figure S1.** Confocal microscopy of GFP and YFP expression in retinas from CD11c and/or CX3CR1 promoters in transgenic mice. **(A)** GFP^hi^ cells present in the outer plexiform layer of naïve CD11c^DTR/GFP^ retina. **(B)** Detection of YFP^hi^ cells in the outer plexiform layer of naïve CX3CR1^YFP-creER^ retina. **(C)** Double transgenic CD11c^DTR/GFP^:CX3CR1^YFP-creER^ mice demonstrated YFP and GFP co-expression in some cells in the outer plexiform layer of naïve retina. Note the cell in the lower right that is both YFP^+^ and GFP^+^. **(D and E)** Perivascular GFP^hi^ cells in naïve and day 7 post-ONC CD11c^DTR/GFP^ retinas. Green - GFP; Red - Isolectin B_4_. **(F)** GFP^hi^ cells were also found near small vessels in the vascular plexus in the inner plexiform layer. Green - GFP; Red - CD11b; Blue - Isolectin B_4_.
