# Supplementary material for: The retinal environment induces microglia-like properties in recruited myeloid cells
Source: J Neuroinflammation. 2019 Jul 20;16:151. doi: 10.1186/s12974-019-1546-9 (PMC6642741; doi:10.1186/s12974-019-1546-9)
Supplement: Supplementary file 2 — Figure S2 Analysis of retinas for local progenitors following ONC. (A) Transient appearance of CD34+ cells in the retinas of non-ablated, non-chimeric CD11cDTR/GFP mice after ONC. CD34+ cell numbers (mean ± SD, n = 4) were determined by direct counts using fluorescence microscopy. (B, C) Immunofluorescence staining showing CD34+ cells in the ganglion cell layer of the retina at 4 days post-ONC. (D) Analysis of bone marrow cells for CD34 expression. (E) Analysis of a non-chimeric B6 mouse 4 days post-ONC revealed the occasional group of CD117+ cells in the inner plexiform layer. (F) Analysis of fetal liver cells for CD117 expression. (DOCX 1308 kb) [file 12974_2019_1546_MOESM2_ESM.docx]

**
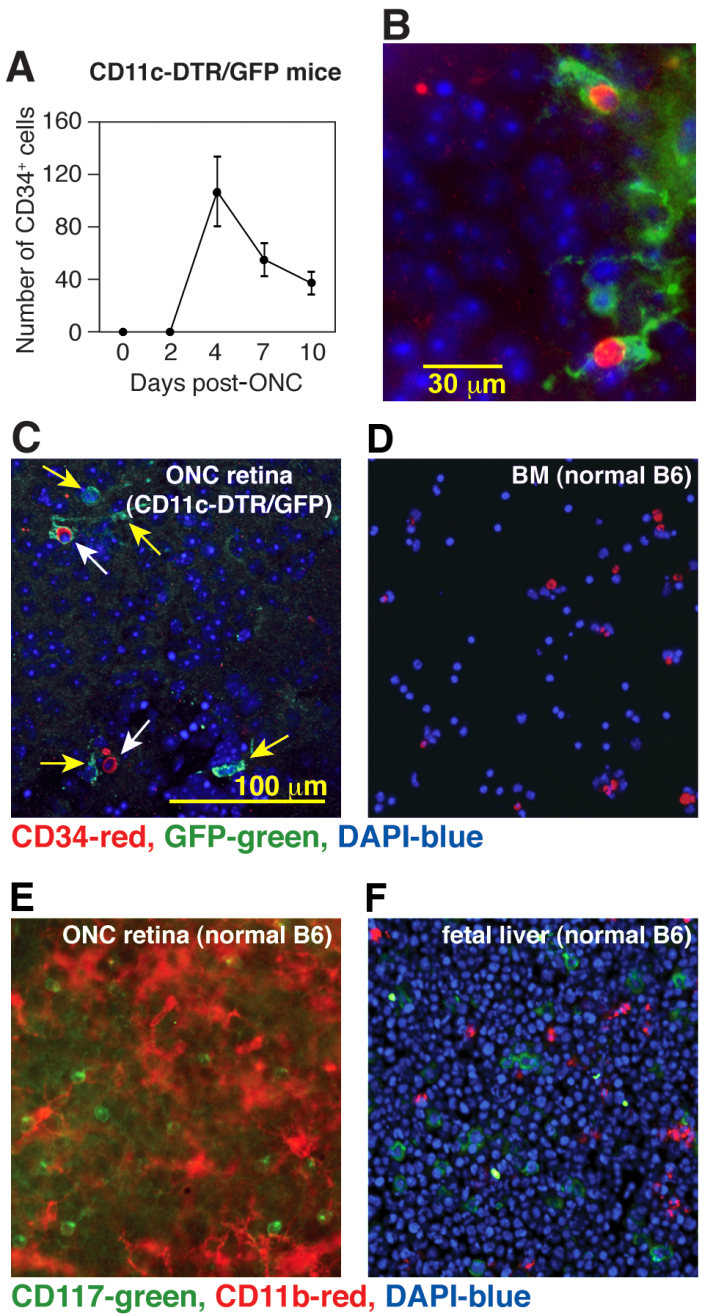
**

**Additional File 2, Figure S2.** Analysis of retinas for local progenitors following ONC. **(A)** Transient appearance of CD34^+^ cells in the retinas of non-ablated, non-chimeric CD11c^DTR/GFP^ mice after ONC. CD34^+^ cell numbers (mean ± SD, n = 4) were determined by direct counts using fluorescence microscopy. **(B, C)** Immunofluorescence staining showing CD34^+^ cells in the ganglion cell layer of the retina at 4 days post-ONC. **(D)** Analysis of bone marrow cells for CD34 expression. **(E)** Analysis of a non-chimeric B6 mouse 4 days post-ONC revealed the occasional group of CD117^+^ cells in the inner plexiform layer. **(F)** Analysis of fetal liver cells for CD117 expression.
